# The Effects of 7 Days of Feeding Pulse-Based Diets on Digestibility, Glycemic Response and Taurine Levels in Domestic Dogs

**DOI:** 10.3389/fvets.2021.654223

**Published:** 2021-05-05

**Authors:** Chloe Quilliam, Yikai Ren, Tressa Morris, Yongfeng Ai, Lynn P. Weber

**Affiliations:** ^1^Department of Veterinary Biomedical Sciences, Saskatoon, SK, Canada; ^2^Department of Food and Bioproduct Sciences, University of Saskatchewan, Saskatoon, SK, Canada

**Keywords:** digestibility, taurine, glycemic response, pulses, canine, fiber

## Abstract

Grain-based carbohydrate sources such as rice comprise 30–50% of commercial pet foods. Some pet foods however have removed the use of grains and have instead incorporated pulses, such as peas and lentils, resulting in grain-free diets. The hypothesis was dog diets with higher levels of dietary fiber will produce a low glycemic response due to decreased rates of digestion and lowered bioavailability of all macronutrients and increased fecal bile salt excretion. This in turn was hypothesized to produce lower plasma concentrations of cysteine, methionine and taurine after 7 days of feeding each test diet in dogs. Six diets were formulated at an inclusion level of 20% available carbohydrate, using white rice flour (grain) or whole pulse flours from smooth pea, fava bean, red lentil or 2 different wrinkled pea varieties (CDC 4,140–4 or Amigold) and fed to beagles in a randomized, cross-over, blinded design. After 7 days feeding each diet, fasting blood glucose was the lowest in the lentil (3.5 ± 0.1 mmol/L) and wrinkled pea (4,140–4; 3.6 ± 0.1 mmol/L) diet periods, while peak glucose levels was lowest after feeding the lentil diet (4.4 ± 0.1 mmol/L) compared to the rice diet. Total tract apparent digestibility of all macronutrients as well as taurine differed among diets yet plasma taurine was not outside normal range. Decreased macronutrient and amino acid digestibility was associated with increasing amylose and dietary fiber content but the specific causative agent could not be determined from this study. Surprisingly, digestibility decreases were not due to increased bile salt loss in the feces since increasing dietary fiber content led to decreased fecal bile salt levels. In conclusion, although pulse-based canine diets have beneficial low glycemic properties, after only 7 days, these pulse-based diets decrease macronutrient and amino acid digestibility. This is likely related at least in part to the lower animal protein content, but on a long-term basis could put domestic dogs at risk for low taurine and dilated cardiomyopathy.

## Introduction

The global pet food industry has been steadily growing and is projected to reach a value of $91 billion USD by 2022 (1). In a majority of homes with pets, pet owners feed them commercially prepared diets as they are affordable and nutritionally complete with the belief that they promote animal health (2). Pet diets in North America are formulated to meet nutritional requirements based on the standards set by the Association of American Feed Control Officials (AAFCO).

Pet owners have an active role in choosing diets to feed their pets and generally make their decisions based on: (i) true knowledge of what comprises a healthy diet, (ii) perception of nutritional requirements, (iii) human diet trends and (iv) overall opinions of the pet food industry. Importantly, owners will often change their pet's diet to match the diet consumed by the human owner (3). Pet foods are marketed strategically to owners, with trendy claims such as “organic,” “natural” and “grain-free” often found with premium pet foods (4). Grain-free diets exclude the use of grains such as wheat, corn, or rice flours and instead incorporate pulses such as peas, lentils and fava beans as the major carbohydrate source (5). While pulses are common dietary ingredients for both humans and animals, they are considered to be highly nutritious primarily due to their high levels of protein, in addition to carbohydrate, fiber, vitamins and minerals (6). Pulse crops are slowly digested due to relatively high amylose, resistant starch and dietary fiber content (7, 8). This characteristic of pulses can be utilized to optimize satiety through a lowered glycemic response and glycemic index (6), a feature that is also in dogs [(9, 10), Briens et al., unpublished].

Postprandial blood glucose responses can vary based on the type of carbohydrate and the rate of digestion (11). In dogs, diets with different levels of starch cause variations in postprandial glucose responses (12). In human studies, foods with a low glycemic index, such as legumes or pulses, are slowly digested, which provides a slow and sustained release of glucose into the blood stream (11). Alternatively, foods that are quickly digested, such as white bread, have a high glycemic index, which result in rapid and high postprandial blood glucose levels (11). Similarly, in both dogs and cats, legumes result in a slow or negligible postprandial glucose response while grains result in a faster, higher postprandial blood glucose response [(9, 13), Briens et al., unpublished]. Thus, pulse-containing diets promote glucose control, insulin sensitivity, satiety, weight control and longer-term health in humans and dogs (10, 14).

While high-protein pulses can decrease the cost to produce a high protein pet food due to a lower need for animal protein, a downside to pulses is that plant protein lacks taurine. Moreover, compared to cereal grains, pulses contain limited amounts of the non-essential amino acid cysteine and reduced amounts of the essential amino acids, methionine and tryptophan (6). Cysteine and methionine are used by the dog liver and the central nervous system to synthesize taurine via the transsulfuration pathway. Thus, taurine is not considered to be an essential amino acid in dogs. While dilated cardiomyopathy is common in dogs (15), some cases are associated with low plasma taurine and can be reversed with taurine supplementation (16). Canine dilated cardiomyopathy was reported by the US Food and Drug Administration (FDA), based initially on 9 case studies, to be associated with feeding grain-free diets in July 2018. This led to a decrease in grain-free dog food sales, decreased use of pulses in pet food and losses to the pulse-growing agriculture sector. Confusion among veterinarians and pet owners as to whether grain-free diets are healthy for dogs was further exacerbated by a recent acknowledgment by the FDA in November 2020 that causes of dilated cardiomyopathy in dogs may be more complicated than just a single ingredient such as pulses and is instead likely multi-factorial. To begin to address some of these questions, this study aimed to explore whether grain-free diets lead to taurine deficiency and if this is associated with simultaneous low cysteine and methionine levels. Moreover, mechanisms by which pulses could deplete taurine, cysteine or methionine require experimental confirmation.

One possible explanation is dietary fiber which was linked to decreased taurine levels in dogs (17, 18). Fiber increases the excretion of fecal bile acids, and since taurocholate is the most common bile salt in dogs, this leads to increased fecal loss of taurine (19, 20). In addition to taurine loss, high dietary fiber decreases protein digestibility, resulting in decreased cysteine and methionine (precursors of taurine) bioavailability. Again, while the slow carbohydrate digestibility of pulses has some beneficial health effects such as low glycemic index, high dietary fiber of pulses may exacerbate the already low taurine, cysteine and methionine found in grain-free diets (20, 21). What is unclear is what component of dietary fiber is responsible for the increased fecal bile salt loss, decreased protein digestibility and decreased sulfur amino acid bioavailability. As a first exploration, this study aims to use diets with increasing levels of dietary fiber and amylose content using different pulses as well as to explore its role in these processes in dogs.

The hypothesis was that dog diets with higher levels of dietary fiber will produce a low glycemic response due to decreased rates of digestion and lowered bioavailability of all macronutrients and increased fecal bile salt excretion. This in turn is hypothesized to produce lower plasma concentrations of cysteine, methionine and taurine after 7 days of feeding each test diet in dogs. In order to investigate these hypotheses, whole and complete diets formulated to include 20% available carbohydrate using a grain (white rice flour) compared to whole pulse flours from smooth pea (CDC Inca), fava bean (CDC Snowdrop), red lentil (CDC Maxim) or 2 different wrinkled pea varieties (CDC 4,140–4 or Amigold) were fed to beagles in a randomized, cross-over, blinded design. After 7 days of feeding each diet, macronutrient digestibility and glycemic responses were examined along with plasma concentrations of cysteine, methionine, and taurine as well as fecal bile salt concentrations in beagle dogs.

## Experimental Methods

All procedures and handling of the dogs were conducted following protocols approved by the University of Saskatchewan's Animal Research Ethics Board according to guidelines that were established by the Canadian Council on Animal Care (Animal Utilization Protocol #20190055).

### Animals

Adult Beagle dogs (*n* = 8; 4 spayed females, 4 neutered males; 8.87 ± 0.90 kg, 2–4 years old) were obtained from certified scientific breeders (Marshal Bioresources, North Rose, NY, USA and King Fisher International, Stouffville, Ontario, Canada). Beagles were housed at the Animal Care Unit (ACU) in the Western College of Veterinary Medicine at the University of Saskatchewan, Saskatoon, SK, Canada. Beagles were group-housed during the day in a large enclosure to allow for daily socialization but were individually kenneled during feedings and overnight. Dogs were walked and socialized on a daily basis. The Beagles were also provided regular health examinations, deworming and routine vaccinations from certified veterinarians to ensure optimal health.

### Diets

The test diets included one control (rice; a grain-containing diet) and five pulse-based diets [all grain-free diets: smooth pea (CDC Inca), wrinkled pea (4,140–4 variety), wrinkled pea (Amigold variety), red lentil (CDC Maxim) and fava bean (CDC Snowdrop)]. All diets were formulated at 20% available carbohydrate using locally obtained flours (flour proximate analyses shown in Supplementary Table 1). A non-digestible Celite marker was also incorporated at 1% for determination of total tract apparent digestibility and measured as acid-insoluble ash in proximate analyses of diet and feces (22). Diets were formulated using the software Creative Concept 5 (Creative Formulation Concepts, Pierz, MN, USA) and were structured to meet the nutritional requirements for canine adult maintenance (see Table 1 for formulations). These requirements were based on AAFCO and the National Research Council recommendations. Feed ingredients were sourced from local and commercial sources as needed and diets were extruded into a dry kibble format using a laboratory-scale, co-rotating, twin-screw extruder (Baker Perkins Ltd, Peterborough, UK) at the University of Winnipeg (Food Science Laboratory, Winnipeg, MB, Canada). All diets were extruded under the same conditions as described in Supplementary Table 2. Diets were then vacuum coated with fat at the Canadian Feed Research Centre (North Battleford, SK, Canada). Samples of all diets were then sent to analytical laboratories for proximate and amino acid analysis (Central Testing Laboratory Ltd., Winnipeg, MB, Canada) according to AOAC standards (23). Dry matter was determined by oven-drying the sample and crude protein determined using the Kjeldahl method while non-fiber carbohydrate and fat were determined through acid-hydrolysis solvent extraction. Metabolizable energy (ME) content of diets was determined through calculation (see footnote to Table 2 for equation). Fiber analyses were performed according to the AOAC 2011.25 method (Eurofins, Toronto, ON, Canada).

**Table 1:** Formulation of test diets with increasing fiber and amylose content listed from left to right. All diets were formulated to include 20% available carbohydrate.

	**Rice diet**	**Lentil diet**	**Smooth pea diet**	**Fava bean diet**	**Wrinkled pea diet (4,140-4)**	**Wrinkled pea diet (Amigold)**
Flour	23.12	42.19	41.67	46.40	58.65	58.14
Chicken By-Product Meal	37.83	21.49	24.26	17.53	8.85	9.03
Cellulose	15	14	12	14	10	10
Chicken Fat^*^	10	10	10	10	10	10
Fish Meal	5	5	5	5	5	5
Canola Oil	6.55	5.00	5.00	5.00	5.00	5.00
Celite	1	1	1	1	1	1
Vitamin/Mineral Premix	1	1	1	1	1	1
NaCl	0.3	0.3	0.3	0.3	0.3	0.3
Choline Chloride	0.1	0.1	0.1	0.1	0.1	0.1
Calcium Carbonate	0.05	0.05	0.05	0.05	0.05	0.05
Dicalcium Phosphate	0.05	0.05	0.05	0.05	0.05	0.38

*All values are expressed as % inclusion as fed*.

* *Included addition of antioxidant Naturox (Kemin, Des Moines, IO USA)*.

**Table 2:** Proximate analyses of test diets after extrusion and fat coating.

	**Rice diet**	**Lentil diet**	**Smooth pea diet**	**Fava bean diet**	**Wrinkled pea diet (4,140–4)**	**Wrinkled pea diet (Amigold)**
DM (%)	89.78	90.43	89.90	90.62	89.33	89.69
Crude Protein (%)^a^	33.69	30.96	32.86	30.41	27.64	26.13
Crude Fiber (%)^b^	6.050	5.230	6.460	8.930	8.770	9.620
Fat (%)^c^	19.79	19.99	16.01	18.45	17.81	19.05
Ash (%)^d^	8.960	7.330	8.420	7.170	6.180	6.500
Metabolizable Energy (kcal/kg)^e^	3,913	4,121	3,806	3,855	3,920	3,856

*Diets are listed in order of increasing fiber and amylose content from left to right. DM, dry matter; All % values are relative to dry matter except Metabolizable Energy (kcal/kg)*.

a *Determined using a Nitrogen/Protein Analyzer (CN628, LECO Corporation, St. Joseph, MI, USA), with a conversion factor of 6.25*.

b *Determined by Central Testing Laboratory Ltd. (Winnipeg, Manitoba, Canada), following Crude Fiber Method by Ankom Technology (2017)*.

c *Determined by Central Testing Laboratory Ltd. (Winnipeg, Manitoba, Canada), following AOCS Method Am 5-04*.

d *Determined by Central Testing Laboratory Ltd. (Winnipeg, Manitoba, Canada), following AOAC Method 942.05*.

e Determined using the ME equation for swine: (kcal/kg)=4151-(122^*^Ash) + (23^*^Crude Protein) + (38^*^Fat)-(64^*^Crude fiber)^*^[1.003-(0.0021^*^ Crude Protein)].

Dogs were fed twice daily, weighed weekly and body condition scored using a 9-point scale (24). During the pre-trial phase of at least a month, each dog was fed a standard commercial diet (Purina Proplan, Mississauga, ON Canada) and individual food portion/maintenance energy required to maintain ideal weight (body condition score of 4–5 on a 9-point scale) determined. Once on trial, isocaloric test diet portions to that determined for each dog in the pre-trial phase was calculated and used throughout the feeding trial without any further adjustment to portion size.

### Glycemic Index & Digestibility Testing

To establish both glycemic responses and starch digestibility of diets as well as the effects on circulating amino acid and taurine levels, Beagles were fed each test diet for 7 days. This was done in a randomized, cross-over, blinded, repeated measures design study with a 3-day washout period on the commercial diet between each test diet and another 7-day feeding period followed by 3-day washout repeated until all diets had been tested in each dog. Total tract apparent digestibility was determined in feces collected on the sixth and seventh day of each feeding period. After collection, feces were kept at−20°C until they were dried at 65°C for 72 h. Fecal samples were then sent to an analytical laboratory to assess nutrient excretion (Central Testing Laboratory Ltd., Winnipeg, MB, Canada). In addition, another portion of dried feces was used to conduct total bile acid analyses using a commercial kit according to manufacturer instructions (Total Bile Acid Assay Kit, Cell Biolabs Inc. San Diego, California, USA) which uses a colorimetric enzyme driven reaction in which bile acids are incubated in the presence of 3-alpha hydroxysteroiddehydrogenase and thio-NADH.

Total tract apparent digestibility was calculated using the formula:

$$\begin{array}{l} Apparent\;Digestibility\\ =\left\{1-\left(\frac{\%\;Nutrient\;in\;Feces\;}{\%\;Nutrient\;in\;Diet}\right)\;x\;\left(\frac{\%\;Indicator\;in\;Diet\;}{\%\;Indicator\;in\;Feces}\;\right)\right\}\\ \times 100 \end{array}$$

On day 7 of feeding, dogs were fasted overnight and 8.0 mL of whole blood was collected the next day from the jugular vein. While still fasted, 5.0 mL of whole blood was collected into EDTA tubes and centrifuged at 2,200 RPM to obtain plasma. Samples were stored at−80°C until assayed for plasma cysteine (measured as the total of the cysteine dimer, cystine, plus the deprotonated form of cysteine called half-cystine), methionine, and taurine at a contract analytical laboratory (Amino Acid Laboratory, University of California Davis, Davis, CA, USA). Beagles were then catheterized in the cephalic vein and fasting blood glucose was determined using an Ultra2 glucometer (OneTouch, LifeScan Canada ULC, Malvern, PA, USA). Depending on the diet fed during the week, dogs were either fed glucose (oral glucose tolerance test) providing 1 g/kg body weight of glucose after consumption of the commercial diet or fed a portion of the test diet fed that week to provide 1 g/kg available carbohydrate (1 g/kg divided by % available carbohydrate) according to established protocols in dogs in our group (9). The amount of available carbohydrate in each diet was determined using a commercially obtained test kit (Available Carbohydrate Assay Kit, Megazyme, Bray, Ireland, see Table 2). Available carbohydrate was defined as total digestible starch (TDS) plus maltodextrins, sucrose, D-glucose, D-fructose and lactose. The available carbohydrate method measures glucose liberated *in vitro* after 4 h incubation (AOAC Method 2020.07). Blood glucose levels were monitored using a glucometer over a 5-h time period (time 0, 5, 10, 15, 20, 30, 45, 60, 90, 120, 150, 180, 210, 240, 270, 300) according to methods established in this group (10).

### Sulfur Containing Amino Acid Plasma Analysis

Plasma sample analyses were conducted at a contract analytical laboratory (Amino Acid Laboratory, University of California Davis, Davis, CA, USA). Modified AOAC Official Method 994.12 alternative III was performed according to GLP (taurine and methionine recovery rates were 97–102%). Variances between duplicates were <5%. Cystine results were corrected by multiplying factor of 2 (recovery rate is about 50%).

### Data Handling and Statistics

All data were tested for normality and outliers using the Kolomogorov-Smirnov test, Q-Q plots and box plots. Depending on the normality of the data either a repeated measure, 1-way ANOVA or a repeated measure, 1-way ANOVA on ranked data was then conducted followed by *post-hoc* Tukey's tests if significance was achieved. Differences were considered statistically significant at *p* ≤ 0.05. Values obtained during the oral glucose tolerance test were not used for statistical analysis and were provided strictly for reference or in glycemic index calculations. Principal components analysis was also performed on the six dietary treatment groups (34 variables studied). Factors were reduced to two components and variables studied were reduced to 24 where ≥83% of the variance across the data was explained. Analyses were performed using SigmaPlot 12.0 and Systat 12.0 (Systat Software Inc. San Jose, CA, USA).

## Results

### Proximate and Amino Acid Analysis of Diets

As expected, crude fiber increased in the diets from the lowest rice control diet to the highest wrinkled pea diets (Table 2). Crude fat of the diets ranged from 16.01% to 19.99%, but was unrelated to dietary fiber (Table 2). Similarly, metabolizable energy ranged from 3,806 to 4,121 kcal/kg. In the test diets, crude protein (% dry matter) ranged from 26.13% to 33.69% which was well-above the AAFCO minimum of 18% dietary protein (25). However, dietary protein content slightly decreased with increasing fiber content, as higher levels of pulse ingredients were incorporated (Table 2). All test diets met or exceeded the AAFCO requirements of 0.33% methionine and 0.65% cystine+methionine (25), except the wrinkled pea CDC 4,140–4 diet which had 0.5% cystine+methionine (Table 2). Dietary methionine was highest in the rice diet at 0.84% on a dry matter basis, with all pulse-based diets containing lower methionine to a low of 0.38% for the high fiber CDC 4,140–4 wrinkled pea diet (Table 2). Dietary cystine ranged from a high of 1.01% for the rice diet to a low of 0.38% for the CDC 4,140–4 wrinkled pea diet, similar to the methionine results (Table 2). The grain-containing rice diet also had the highest level of taurine at 0.14% (Table 2). In contrast, the pulse containing diets had lower, varying amounts of taurine which ranged from 0.07% to 0.12% as shown in Table 3.

**Table 3:** Cystine, methionine and taurine levels in test diets after extrusion and fat coating.

	**Rice diet**	**Lentil diet**	**Smooth pea diet**	**Fava bean diet**	**Wrinkled pea diet (4,140–4)**	**Wrinkled pea diet (Amigold)**
Cystine^a^	1.010	0.610	0.170	0.640	0.120	0.490
Methionine^a^	0.840	0.470	0.700	0.480	0.380	0.460
Cystine & Methionine^a^	1.850	1.080	0.870	1.120	0.500	0.950
Taurine^a^	0.140	0.090	0.120	0.090	0.070	0.070
LMWDF^b^	<0.6	2.2	2.0	1.6	4.8	4.9
Soluble HMWDF^b^	0.9	1.3	1.2	1.7	1.5	1.9
Insoluble HMWDF^b^	15.2	18.1	16.7	20.6	21.3	21.8
TDF^b^	16.1	21.6	19.9	23.9	27.6	28.6
Amylose content^c^	5.1 ± 0.2	7.4 ± 0.2	7.6 ± 0.1	7.7 ± 0.1	13.9 ± 0.1	14.6 ± 0.1
Available carbohydrate (g/100g)^d^	23.9 ± 0.6	24.7 ± 0.3	25.2 ± 0.3	23.9 ± 0.3	22.4 ± 0.6	22.7 ± 0.4
Meal size fed to provide 1 g/kg available carbohydrate (g whole diet/kg dog)	4.2	4.0	4.0	4.2	4.5	4.4

*Also shown are the contents of different fiber types, amylose, available carbohydrate and meal size needed to provide 1 g/kg available carbohydrate for each diet. Diets are listed in order of increasing fiber and amylose content from left to right. All values are expressed as % dry matter unless stated otherwise. LMWDF, low molecular weight dietary fiber; HMWDF, high molecular weight dietary fiber; TDF, total dietary fiber*.

a *Determined by Central Testing (Winnipeg, MB, Canada) using UPLC and ninhydrin detection*.

b *Determined by Eurofins (Toronto, ON, Canada) using AOAC 2011.25 dietary fiber method*.

c *Determined using an iodine colorimetric method of (26)*.

d *Determined using Megazyme Available Carbohydrate Assay Kit following AOAC Method 2020.07*.

### Fiber, Amylose and Carbohydrate Content of the Diets

Diets were formulated to contain 20% available carbohydrate and results of the available carbohydrate kit for diets showed good agreement with the target (Table 3). Available carbohydrate content (defined as the amount of glucose liberated by amyloglucosidase+pancreatin in 4 h *in vitro* at 37°C) in the formulated test diets ranged from 22.4% to 25.2%, as shown in Table 3. This available carbohydrate value was then used to calculate what meal size needed to be fed to provide 1 g available carbohydrate per kilogram body weight of the dogs during glycemic testing (Table 3). The same test diets prior to fat coating were tested for *in vitro* starch digestibility using Englyst methodology in separate study by this group (27). Uncoated wrinkled pea diets after extrusion (both Amigold and 4,140–4) had higher resistant starch content (15.1–19.0% on a dry starch basis) compared to the other pulse-based or rice-based diets (2.7–5.5% on a dry starch basis). Also, both wrinkled pea diets had lower gelatinized starch after extrusion (11.9–12.3% dry matter) compared with those of round pea, lentil, fava bean, and rice diets (17.1–19.6%; 9).

Dietary fiber analyses of the fat coated test diets used in this feeding study demonstrated that they had varying levels of dietary fiber (Table 3). Low molecular weight dietary fiber varied from <0.6–4.9% for the rice and the wrinkled pea (Amigold) diets, respectively (Table 3). High molecular weight dietary fiber was subdivided into two categories: insoluble high molecular weight dietary fiber and soluble high molecular weight dietary fiber. The soluble high molecular weight dietary fiber varied from 0.9% to 1.9% (rice and Amigold wrinkled pea diets, respectively), while insoluble high molecular weight dietary fiber varied from 15.2% to 21.8% (rice and Amigold wrinkled pea diets, respectively; Table 3). Similarly, total dietary fiber ranged from 16.1% to 28.6% (rice and Amigold wrinkled pea diets, respectively; Table 3). Finally, the wrinkled pea (Amigold) pulse-containing diet had the highest amylose content (14.6%) while the rice-containing diet had the lowest level of amylose at 5.1% (Table 3).

### Digestibility

Total tract apparent digestibility of crude protein varied across the diets (ANOVA, *p* < 0.001). Crude protein digestibility varied from 72.95% to 84.35%, the rice diet had the highest crude protein digestibility, and the wrinkled pea (Amigold) diet had the lowest (Table 4). For sulfur-containing amino acids, the total tract apparent digestibility of cystine, methionine, cystine+methionine and taurine varied among test diets (ANOVA, *p* < 0.001 for all but taurine where *p* = 0.034; Table 4). Variability was noted for cystine digestibility which ranged from 6.5% to 90% (Table 4). This variability was not due to diarrhea. Although fecal output and score (quality) were not quantitated in this study, qualitatively, no obvious changes were observed among diets. All other macronutrient (fat and starch) digestibility values were also different among diets (Table 4).

**Table 4:** Total tract apparent digestibility analyses of protein, fat, starch, cystine, methionine, cystine+methionine and taurine in the 6 different test diets formulated at 20% available carbohydrate with variable amounts of fiber, fed for 7 days.

	**Rice**	**Lentil**	**Smooth pea**	**Fava bean**	**Wrinkled pea (4,140–4)**	**Wrinkled pea (Amigold)**	***P*-Value**
Protein**	84.35 ± 1.3^b^	81.49 ± 0.69^b^	80.85 ± 0.66^b^	79.21 ± 0.67^a, b^	78.90 ± 0.58^a, b^	72.95 ± 1.37^a^	<0.001
Fat*	98.47 ± 0.18^c^	97.52 ± 0.30^b, c^	96.40 ± 0.65^a, b^	97.52 ± 0.27^b, c^	96.85 ± 0.42^a, b^	95.82 ± 0.48^a^	<0.001
Starch**	97.71 ± 0.41^b, c^	97.72 ± 0.76^c^	97.77 ± 0.23^a, b, c^	98.28 ± 0.20^b, c^	89.68 ± 2.08^a, b^	87.74 ± 2.49^a^	<0.001
Cystine*	89.76 ± 0.78^d^	84.91 ± 1.09^d^	41.80 ± 2.30^b^	86.10 ± 0.78^d^	6.52 ± 4.14^a^	73.31 ± 3.45^c^	<0.001
Methionine**	79.22 ± 1.66^b^	76.27 ± 0.85^a, b^	83.29 ± 0.99^b^	75.80 ± 0.82^a, b^	64.72 ± 2.20^a^	53.56 ± 5.24^a^	<0.001
Cystine + Methionine**	84.98 ± 1.01^c^	80.97 ± 0.94^b, c^	75.18 ± 1.08^a, b^	81.85 ± 0.73^b, c^	41.12 ± 6.47^a^	63.75 ± 4.07^a, b^	<0.001
Taurine**	82.92 ± 1.83^a, b^	80.78 ± 11.58^b^	77.44 ± 3.09^a, b^	69.98 ± 2.19^a^	77.57 ± 5.96^a, b^	74.42 ± 5.11^a, b^	0.034

Diets are listed in order of increasing fiber and amylose content from left to right. All values are expressed as % of total tract apparent digestibility. Data is shown as Mean ± SEM; n = 8 dogs. Different letters indicate significant differences among diets using Tukey's post-hoc analysis (P < 0.05) after significant P-values reported in 1-Way Repeated Measures ANOVA^*^ and Friedman's One-Way ANOVA on Ranked Data^**^. Where same letters are indicated in a row, no significant differences among diets were detected.

*Protein, and fat, determined by Central Testing (Winnipeg, MB Canada) as described in Table 2. Diet and fecal amino acids and starch were determined by Central Testing (Winnipeg, MB, Canada) using UPLC with ninhydrin detection and enzymatically using UV detection, respectively*.

### Glycemic Response

**Figure 1:**
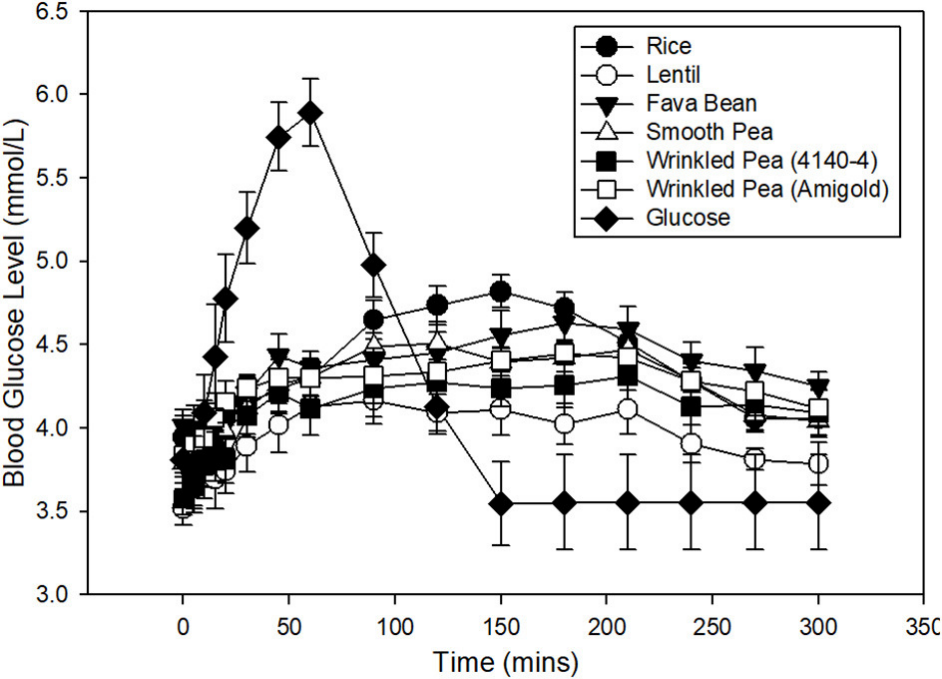
Time course of blood glucose response in fasted dogs after oral glucose challenge (1 g glucose/kg body weight) after 7 days of feeding a commercial husbandry diet (filled circles). Time course of blood glucose response in fasted dogs after consuming a meal of each test diet (1 g available carbohydrate/kg body weight) is also shown. Dogs were fed each test diet formulated at 20% available carbohydrate with increasing levels of fiber and amylose (lowest to highest from top to bottom in figure legend) for 7 days prior to an overnight fast, followed by the test meal the next day. Data is shown as Mean ± SEM; *n* = 8 dogs.

Blood glucose increased from fasting after feeding glucose or a meal with 1 g available carbohydrate/kg body weight, then returned to baseline within 5 h in dogs (Figure 1). From these glycemic response figures, quantitative data and statistical analyses on peak, time to peak, area under the curve and glycemic index were calculated (Table 5). In dogs fed the commercial diet for 7 days, the fasting blood glucose level prior to the oral glucose tolerance test was 3.8 ± 0.2 mmol/L. After 7 days of feeding the test diets, fasting blood glucose had variation with diet in these same dogs (Figure 1; ANOVA, *p* < 0.001; Table 5). After ingestion of the glucose standard, glucose levels peaked at 52.5 ± 5.5 min with a blood glucose value of 6.3 ± 0.2 mmol/L (Figure 1, Table 5). Time to peak blood glucose was longer after a meal of a whole diet and the peak was lower when compared to the response to the glucose standard (Figure 1).

**Table 5:** Quantitative measures of glycemic response in fasted beagles after feeding a meal of different test diets.

	**Glucose**	**Rice**	**Lentil**	**Smooth pea**	**Fava bean**	**Wrinkled pea (4,140-4)**	**Wrinkled pea (Amigold)**	***P*-Value**
Fasted Blood Glucose (mmol/L)*	3.8 ± 0.2	3.9 ± 0.1^b^	3.5 ± 0.1^a^	3.8 ± 0.06^a^	4.0 ± 0.1^b^	3.6 ± 0.1^a^	3.8 ± 0.1^b^	<0.001
Peak (mmol/L)*	6.3 ± 0.2	5.0 ± 0.09^b^	4.4 ± 0.1^a^	4.7 ± 0.1^a, b^	4.8 ± 0.1^a, b^	4.5 ± 0.1^a, b^	4.7 ± 0.1^a, b^	0.01
Time to Peak (min)**	52.5 ± 5.5	135 ± 12.7^a^	99.4 ± 18.6^a^	91.9 ± 9.2^a^	132.5 ± 20.9^a^	111.6 ± 18.0^a^	130 ± 22.8^a^	0.2
AUC(mmol/L x min)*	849.3 ± 19.6	810.9 ± 15.8^b^	726.5 ± 21.7^a^	780.2 ± 14.0^a, b^	792.6 ± 12.6^a, b^	749.4 ± 18.8^a, b^	776 ± 14.1^a, b^	0.02
Glycemic Index*		95.7 ± 2.2^c^	85.8 ± 2.8^a^	92.2 ± 2.6^b, c^	93.6 ± 2.2^b, c^	88.3 ± 1.5^a, b^	91.5 ± 1.2^b, c^	<0.001

*Diets are listed in order of increasing fiber and amylose content from left to right. Data is taken from that shown in Figure 1*.

Data is shown as Mean ± SEM; n = 8 dogs. Different letters indicate significant differences among diets using Tukey's post-hoc analysis (P < 0.05) after significant P-values reported in 1-Way Repeated Measures ANOVA* and Friedman's One-Way ANOVA on Ranked Data**. Where same letters are indicated in a row, no significant differences among diets were detected. Glucose data was used to calculate glycemic index and is shown for reference, but was not used in the statistical analyses of the diets.

Dogs fed the lentil-based diet and wrinkled pea (4,140–4) diet for 7 days had the lowest fasting blood glucose levels (3.5 ± 0.1 mmol/L) and (3.6 ± 0.1 mmol/L), respectively (Table 5). The peak blood glucose was also different among diets (ANOVA, *p* = 0.01), while the time to peak was not different (ANOVA, *p* = 0.20). Dogs fed the rice diet had the highest peak blood glucose at 5.0 ± 0.09 mmol/L, while the lowest peak blood glucose was observed with the lentil diet at 4.4 ± 0.1 mmol/L (Table 5). The area under the blood glucose response curve (AUC) was different among diets (ANOVA, *p* = 0.02; Table 5), ranging from 810.9 ± 15.8 mmol/L x min for the rice diet to a low of 726.5 ± 21.7 mmol/L x min for the lentil diet (Table 5). Glycemic index values of the diets followed the AUC trend, with differences among diets (ANOVA, *p* < 0.001; Table 5). The rice diet had the highest glycemic index of 95.7 ± 2.2, while the lowest glycemic index was observed in the lentil diet at 85.8 ± 2.8 (Table 5).

### Sulfur Containing Amino Acids in Plasma

After 7 days of feeding each diet, fasting plasma taurine was different in the dogs after feeding different diets (ANOVA, *p* = 0.021; Table 6). Feeding rice and the lentil diets produced the highest plasma taurine levels in the dogs at 99 and 111 nmol/ml (equivalent to μmol/L), while feeding the fava bean diet produced the lowest plasma taurine at 73 nmol/ml (Table 6). Despite differences in dietary content of cystine and methionine (Table 2), after 7 days of feeding each diet, fasting levels of plasma methionine and half-cystine were not significantly different (Table 6). In contrast, plasma cysteine varied greatly among the dogs fed the different diets with the highest levels when the lentil and rice diets were fed.

**Table 6:** Plasma amino acid levels of taurine, half-cystine, cysteine and methionine observed in fasted dogs after 7 days of feeding each test diet.

	**Rice**	**Lentil**	**Smooth pea**	**Fava bean**	**Wrinkled pea (4,140-4)**	**Wrinkled pea (Amigold)**	***P*-Value**
Half-cystine*	17.61 ± 0.85^a^	19.16 ± 1.62^a^	17.46 ± 1.14^a^	16.43 ± 1.42^a^	18.34 ± 1.59^a^	17.13 ± 1.12^a^	0.42
Cysteine**	132.5 ± 32.79^a^	2732.75 ± 139.75^b^	807.38 ± 279.88^a, b^	458.63 ± 213.27^a^	158.88 ± 213.27^a^	147.86 ± 28.58^a^	0.002
Methionine*	56.31 ± 3.13^a^	50.18 ± 2.09^a^	53.58 ± 2.08^a^	54.74 ± 2.51^a^	53.35 ± 3.13^a^	52.55 ± 3.54^a^	0.52
Taurine**	99.03 ± 11.90^a, b^	111.14 ± 18.04^b^	82.24 ± 8.67^a, b^	72.68 ± 12.74^a^	86.63 ± 10.95^a, b^	89.74 ± 15.36^a, b^	0.021

*Diets are listed in order of increasing fiber and amylose content from left to right*.

*All values expressed nmol/ml (equivalent to μmol/L). Amino acids were measured at a contract analytical laboratory (Amino Acid Laboratory, University of California Davis, Davis, CA, USA) using modified AOAC Official Method 994.12 alternative III*.

Data is shown as Mean ± SEM; n = 8 dogs. Different letters indicate significant differences among diets using Tukey's post-hoc analysis (P < 0.05) after significant P-values reported in 1-Way Repeated Measures ANOVA^*^ and Friedman's One-Way ANOVA on Ranked Data^**^. Where no letters are indicated in a row, no significant differences among diets were detected.

### Bile Acid Assay

**Figure 2:**
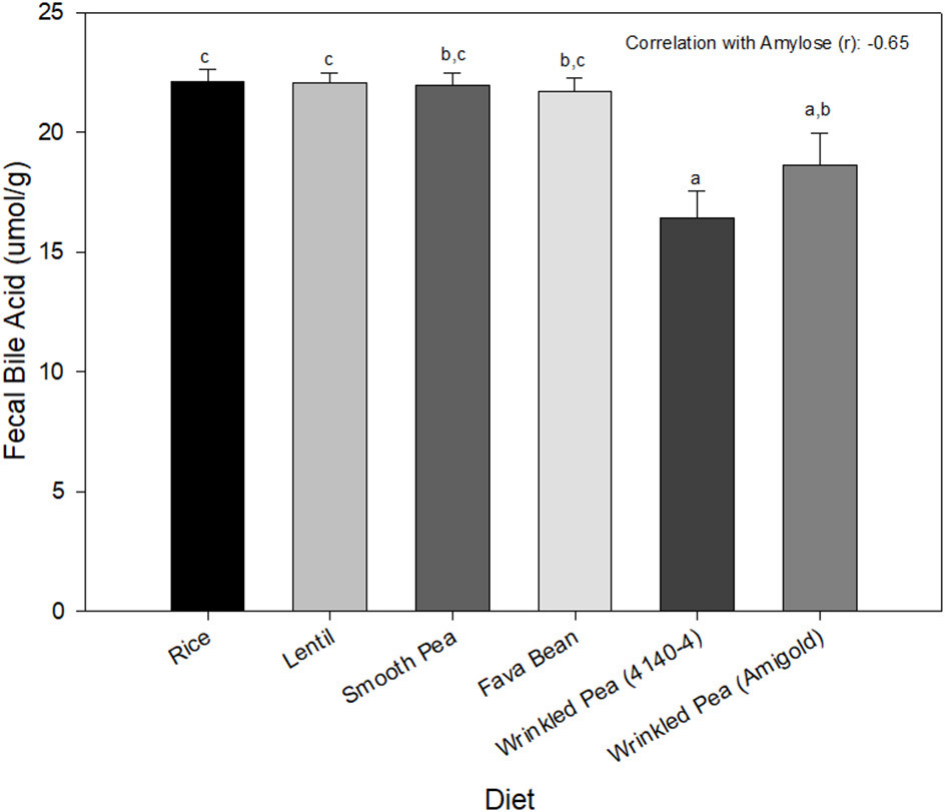
Fecal bile acid content from dogs after 7 days of feeding each test diet. Diets are listed in order of increasing fiber and amylose content from left to right. Data is shown as Mean ± SEM; *n* = 8 dogs. Different letters indicate significant differences using Tukey's *post-hoc* analysis (*P* < 0.05) after 1-Way Repeated Measures ANOVA.

Fecal bile acid content was significantly different among diets, with the rice diet having the highest value and the wrinkled pea 4,140–4 diet having the lowest (Figure 2).

### Principal Components Analysis

**Figure 3:**
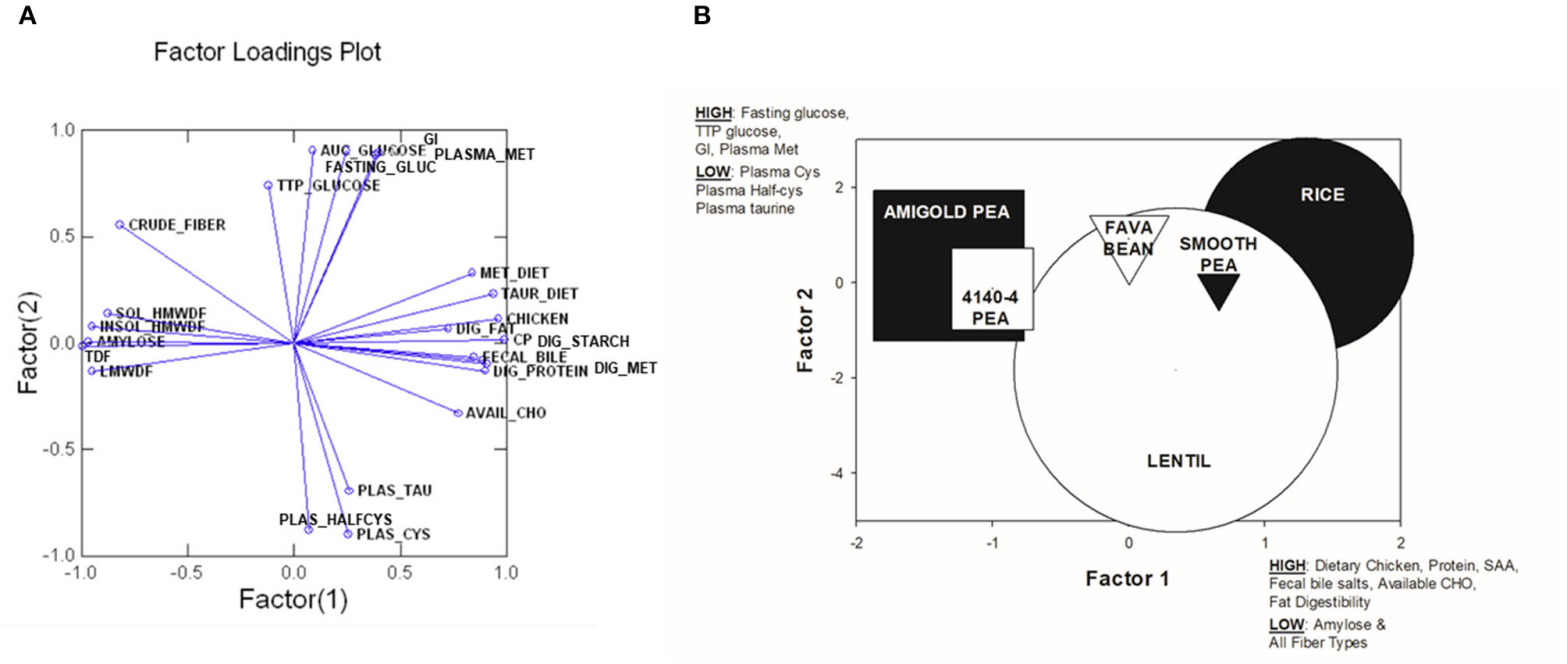
Plots from Principal Components Analysis of the top 24 variables that explained the most variability among diets. **(A)** Factor loadings plot for factor 1 and factor 2. **(B)** A plot of weighted factor scores for components 1 vs. components 2 for each test diet in this study from the Principal Components Analysis. The size of the shape indicates the 95% confidence intervals and distance between shapes indicates greater difference among diets. Higher, positive scores for Factor 1 were associated with high dietary chicken content, dietary crude protein, dietary sulfur amino acid content (SAA; cysteine, methionine and taurine), fecal bile salts, dietary available carbohydrate and fat digestibility, but low amylose and all fiber types. In contrast, a high positive score for Factor 2 was associated with high fasting blood glucose, long time to peak blood glucose, high glycemic index and high plasma methionine, but low plasma cysteine, plasma half-cysteine and plasma taurine. SOL_HMWDF, soluble high molecular weight dietary fiber; INSOL_HMWDF, insoluble high molecular weight dietary fiber; TDF, total dietary fiber; LMWDF, low molecular weight dietary fiber; TTP_GLUCOSE, time to peak glucose; FASTING_GLUC, fasting blood glucose; AUC_GLUCOSE, area under the curve for blood glucose response; GI, glycemic index; PLASMA_MET, plasma methionine; MET_DIET, dietary level of methionine; TAUR_DIET, dietary level of taurine; DIG_FAT, total tract apparent digestibility of fat; DIG_STARCH, total tract apparent digestibility of starch; FECAL BILE, fecal bile salts; DIG_PROTEIN, total tract apparent digestibility of protein; DIG_MET, total tract apparent digestibility of methionine; AVAIL_CHO, available carbohydrate in diet; PLAS_TAU, plasma taurine; PLAS_HALFCYS, plasma half-cysteine; PLAS_CYS, plasma cystine.

In order to assess the relationships among the variables tested, a Principal Components Analysis (PCA) was run initially with all variables measured in all 6 test diets. The top 24 variables that were correlated to the first two components were retained in the final PCA analysis. These 24 variables explained 83.13% of variance in this final analysis (see Table 7). A factor loadings plot of these variables (Figure 3A) clustered variables with the greatest tendency to be positively related to each other. A plot of weighted factor scores for each of these 24 variables for each diet resulted in a graph where the rice diet was clearly separated from the two wrinkled pea diets, while fava bean, smooth pea and lentil diets were intermediate (Figure 3B). This confirms the order we predicted based on fiber and amylose content. In support of this, the greatest separation among the diets was along the x-axis showing Factor 1 scores (Figure 3B) where high positive scores indicated diets with high dietary content of chicken meal, crude protein, sulfur amino acids (cystine, methionine and taurine) as well as high fecal bile salt content, available carbohydrate and fat digestibility, but low values for dietary content of amylose and all fiber fractions (Figure 3B). The diets did not separate as much on the y-axis except for the lentil diet, but the 95% confidence intervals were very large for this diet and it did overlap with all diets on this axis (Figure 3B). High positive y-axis values for Factor 2 were associated with high fasting blood glucose, long time to peak blood glucose, high glycemic index and high plasma methionine values, but low plasma cystine, half-cysteine and taurine values.

**Table 7:** Principal Components Analysis of the top 24 variables examined in this study that explained 83% of the variation among diets.

	**Component 1**	**Component 2**
**Total variance explained (%)**	**56.03**	**27.095**
**Variable**	**Component loadings**	
Total Dietary Fiber	−0.992	−0.016
Crude Protein	0.990	0.014
Amylose	−0.968	0.007
Chicken By-Product Meal	0.962	0.115
Insoluble High Molecular Weight Dietary Fiber	−0.950	0.076
Low Molecular Weight Dietary Fiber	−0.949	−0.134
Dietary Taurine	0.938	0.231
Methionine Digestibility	0.909	−0.096
Protein Digestibility	0.901	−0.131
Starch Digestibility	0.889	−0.083
Soluble High Molecular Weight Dietary Fiber	−0.876	0.138
Fecal Total Bile Acids	0.847	−0.067
Dietary Methionine	0.839	0.329
Dietary Crude Fiber	−0.818	0.553
Available Carbohydrate	0.773	−0.331
Fat Digestibility	0.726	0.069
Fasting Blood Glucose Levels	0.244	0.904
Area Under the Curve (Glucose)	0.089	0.903
Plasma Cysteine Levels	0.258	−0.898
Glycemic Index	0.399	0.895
Plasma Methionine Levels	0.385	0.883
Plasma Half-Cystine	0.071	−0.880
Blood Glucose, Time to Peak	−0.118	0.740
Plasma Taurine Levels	0.263	−0.696

*The strength of the relationship (r-value) for the two components, referred to as component loadings, are shown for each of these 24 variables*.

## Discussion

The most important findings of this study were that the pulse-based, grain-free diets produced a lowered glycemic response, which could be utilized to promote increased satiety and decreased risk of diabetes mellitus. This postprandial glycemic response was negatively associated with plasma taurine and cysteine levels, but positively associated with plasma methionine levels in dogs after 7-days of feeding each test diet. Surprisingly, fiber content (all fractions) and dietary amylose content were not strongly related to plasma sulfur amino acid levels. Instead, fiber and amylose were negatively correlated to digestibility of all macronutrients, including sulfur containing amino acids. Despite increasing levels of dietary fiber as amylose content increased among diets, excretion of fecal bile acids in this study unexpectedly decreased. In addition, on a short-term feeding basis (7 days), grain-free diets did not cause a detrimental impact on the plasma taurine status in dogs, despite decreased taurine digestibility. This study showed promising effects of grain-free, pulse-based diets that could be utilized for the improvement of health in dogs.

### Test Diet Properties and Relationship to Fiber Content

All test diets were formulated to be as similar as possible, while aiming to achieve ~20% available carbohydrate. This produced desired variations among the diets regarding their dietary fiber and amylose content. Diets with increasing levels of dietary fiber and amylose were among the diets that incorporated the largest amounts of pulse flour which provided plant protein and subsequently needed decreased amounts of animal protein (chicken meal) to be isonitrogenous. In addition, decreasing the amount of animal protein among diets produced decreasing levels of taurine in diets. Other studies confirmed that seafood and poultry products contained high concentrations of taurine, while plant products do not (28). Dietary fiber also did not appear to have any impacts on the other amino acids studied within the different diets such as cystine, methionine and cysteine + methionine. All pulse diets had lower levels of these amino acids in comparison to the rice diet due to pulses possessing limited content of these amino acids (6, 29). This could explain why there were variations of dietary cystine, methionine and cystine+methionine in the test diets.

In this study increasing amylose content among the different diets was associated with increasing levels of total dietary fiber. This was due to pulses with higher amylose levels containing larger amount of resistant starch, which is the fraction of starch that reaches the colon intact, as it is not digested in the small intestine (30). As reviewed by (30), resistant starch such as amylose is also viewed as dietary fiber, partially explaining the link between increased dietary fiber and amylose among diets. Increased amounts of amylose and dietary fiber influence nutrient digestibility and postprandial glucose responses (13). Thus, based on those diets we produced, our results support our hypothesis that the diets with higher fiber and lower animal-source protein exert negative effects on macronutrient and amino acid digestibility and produce lower glycemic responses. However, after 7-days of feeding diets, all dog plasma levels of cysteine, methionine and taurine were within normal range. Longer term studies are needed to confirm this.

### Effect of Different Pulse-Based Diets on Glycemic Response

The diets for this study were chosen to demonstrate how increasing dietary fiber produced positive attributes on glycemic responses but might have negative impact on other nutrients in dogs. In humans, increased consumption of dietary fiber and amylose decreased postprandial blood glucose (31–34). High amylose pulse crops when used as an ingredient in dog diets minimized the risk of obesity and diabetes mellitus (13) and pulse-based diets have low glycemic properties in dogs [(9, 13, 35), Briens et al., unpublished]. However, a study systematically using a gradient of amylose content in dog diets had not yet been performed. The current study demonstrates that while amylose and total dietary fiber content is clearly an important factor in decreasing glycemic response in dogs, the spurious low results with the lentil diet point likely contributed to the lack of correlation of all the glycemic endpoints to dietary fiber and amylose content.

In addition to dietary fiber content determining glycemic response, other factors include digestion rate, amount of diet ingested, processing factors and dietary composition (13). In this study, all diets had 22–25% total digestible starch. However, since wrinkled pea flour contains much lower starch levels (~34%), the high amylose wrinkled pea diets contained up to 65% flour which contributed a large amount of fiber compared to the low amylose diets. In human studies, resistant starch is an indication of high levels of amylose and contributes to decreased starch digestibility and increased levels of total dietary fiber (36). In similar studies, changes in dietary fiber impacts glycemic response by slowing down the rate of passage of feed and the rate of hydrolysis on polysaccharides in starch (12, 37). Postprandial glucose responses are further impacted by dietary fiber as it is believed to prolong glucose absorption, thus reducing variations in glucose responses (13). Overall, glycemic response in this study was less impacted by the varying levels of amylose and dietary fiber within the different test diets and instead related to some other unidentified factor that was associated with high plasma taurine and cysteine. Further studies are needed to confirm this finding and explore how this happens.

### Effect of Amylose vs. Fiber on Macronutrient and Amino Acid Digestibility

The results of the current study which found decreasing digestibility of all macronutrients (protein, fat and starch) with increasing dietary amylose and total dietary fiber agrees well-with a study conducted by (38). Digestibility of crude protein decreased as total dietary fiber consumption increased in dogs (38). This could be explained possibly by endogenous factors within the pulse flours having intrinsic interactions to form structures with starch, such as amylose, to limit the digestibility of protein (39). Similar to what is seen with protein digestion, lipids also interact with starches, creating a single-helical structure with amylose molecules and limits the enzymatic digestibility of starch (40). These amylose-lipid complexes that are resistant to starch digestion are formed during the exposure to elevated temperatures, which occur during the process of extrusion (41, 42). Due to the possibility of these processes, lipid digestibility in this study decreased as amylose levels and total dietary fiber increased. For example, a study in growing pigs demonstrated that increasing levels of fiber decreased the total tract apparent digestibility of both crude protein and fat (43).

In addition to amylose and dietary fiber impacting the digestibility of macronutrients, they also impact the digestibility of amino acids. In this study increasing levels of amylose and dietary fiber were negatively correlated to digestibility of cystine, methionine and taurine. Diets containing high amounts of dietary fiber not only lead to a greater possibility of sulfur amino acid excretion, but also greater microbial overgrowth and taurine assimilation by the microflora (18). This would be detected as increased total tract apparent digestibility, so is inconsistent with our observations. What this study could not determine is whether a particular fiber fraction or amylose content were driving changes in sulfur amino acid digestibility since all of fiber fractions and amylose were all equally negatively associated with digestibility.

### Effect of Different Pulse-Based Diets on Plasma Levels of Sulfur-Containing Amino Acids

Unlike cats, dogs are able to synthesize taurine from the sulfur-containing amino acids cysteine and methionine (15). Thus, taurine is not considered an essential dietary nutrient for dogs, while methionine and methionine+cysteine have dietary minima in dogs (25). In this study, plasma taurine levels ranged from 73 to 111 nmol/mL in fasted dogs after 7 days of feeding test diets which falls within previously reported taurine reference ranges of 63–194 nmol/mL (16, 44–46). Other studies disagree on whether or not grain-free diets contribute to decreased plasma taurine levels in dogs. Some studies determined that dogs consuming grain-free diets have an increased prevalence of taurine deficiencies (16, 44), while others have noted no change or improvements in taurine status in dogs when consuming grain-free diets (47, 48). Despite lower dietary taurine content with increasing dietary fiber and amylose in the current study, plasma taurine remained within normal range. This could be due to the short-term nature of the current study (7 days per diet). Another important factor linked to a lack of consistent change in plasma taurine may be that taurine levels in target tissues such as the heart are more relevant and these tissue levels could be depleted before plasma levels of these free amino acids fall (17). Moreover, the beagle breed is not predisposed to either taurine deficiencies or dilated cardiomyopathy (19). Taken together, this study demonstrated that short-term consumption of both grain-containing and grain-free diets had no major effect on plasma taurine levels in dogs.

In this study, plasma levels of half-cystine ranged from 16 to 19 nmol/ml while plasma levels of methionine varied from 50 to 56 nmol/mL. Levels for both of these amino acids are lower than, but close to, the value reported in a dog study with 46 and 57 nmol/mL, respectively (46). Plasma cysteine values had much greater and unexpected variation from 132 to 2,732 nmol/mL among diets in the current study. Cysteine numbers are suspect for two reasons. First, cysteine is unstable after sample collection and rapidly forms disulfide bonds with itself to form the dimer called cystine or with other plasma proteins which are subsequently removed before analysis (personal communication from Amino Acid Laboratory, University of California Davis, Davis, CA, USA). Second, in this experiment, the cysteine data was more likely overestimated due to interference during HPLC analysis from L-alpha-aminoadipic acid that co-eluted with cysteine (49). Coupled with the fact that dietary cystine levels did not vary that much, it seems likely that dietary fiber has no effect on plasma cysteine, methionine or taurine levels, at least after 7 days of feeding test diets.

### Effect of Dietary Fiber and Amylose on Fecal Bile Acids

Contrary to the original hypothesis, fecal excretion of total bile acids decreased as dietary fiber and amylose increased in this study in beagles after 7 days of feeding test diets. The findings of this study disagree with reports that dietary fiber can bind bile acids within the intestinal lumen, leading to increased fecal excretion of bile acids (47, 50, 51). However, the results of this study agree with another report that grain-free diets did not lead to an increased excretion of bile acids in dogs (19). Soluble dietary fiber abundant in pulses, was proposed to lower taurine availability in companion animals (18). One of the major roles of taurine in dogs is conjugation with bile acids to form the predominant bile salt, taurocholate (17, 18). While soluble fibers bind bile acids to prevent their reabsorption through the entero-hepatic circulation, thereby lowering lipids (a beneficial health effect), this effect could also deplete taurine via taurocholate loss in the feces, leading to taurine wasting (17). Alternatively, legumes high in dietary fiber can also act as prebiotics for the gut microbiota, changing the microbial population composition and overgrowth, which could enhance taurine degradation in the small intestine before it can be absorbed (45). Future studies need to explore whether feeding periods >7 days cause increased bile salt loss, whether specific classes of bile acids are affected or whether the effects on intestinal microbiota lead to taurine depletion in dogs and if so, which dietary fiber components, if any, contribute to this loss.

## Study Strengths and Limitations

A strength of this study was the detailed glycemic response data for each diet. Another strength of this study was the use of multiple pulses to study if dietary fiber and amylose are impacting variables in different pulses or if there is another component that should be studied. One limitation of the study design was the sample size of eight beagles. However, we combated this limitation by using a cross-over, repeated-measures design, allowing all dogs to rotate through all diets and be studied. Another limitation of this study could be insufficient duration of feeding each diet for plasma sulfur containing amino acids to change. Ongoing studies in this group are exploring effects of longer-term feeding periods but establishing what happens in shorter time frames is also important information. For digestibility measurements, a limitation was the use of total tract apparent digestibility method which was necessary since our studies require minimally invasive, non-lethal techniques to allow our dogs to be adopted into homes once retired. True digestibility can only be assessed through either lethal sampling to remove digesta along the length of the intestinal tract or through ileal sampling that requires surgical creation of a permanently disfiguring ileal cannula.

## Conclusions

Pulses are beneficial at producing a low glycemic response in dogs and higher amylose pulses such as the wrinkled pea (4,140–4) have superior low glycemic properties in dogs. However, even pulses such as red lentil with relatively low amylose and dietary fiber could also be processed to produce a low glycemic diet. The trade-off for beneficial low glycemic properties of high dietary fiber and high amylose pulses is decreased macronutrient and amino acid digestibility. However, this study did not find high fiber or amylose to be associated with increased fecal bile acid secretion and instead observed a decrease. In addition, due to the limitations of diet formulation in this study, high-fiber pulse diets contained less animal-source protein and higher plant-based proteins compared with the rice diet, which could be another important factor leading to decreased digestibility of certain nutrients. However, despite decreased nutrient digestibility, plasma levels of sulfur-containing amino acids, including taurine remained within normal range after 7 days of feeding test diets, suggesting at least in the short term, that the benefits may outweigh any negative nutritional effects of high fiber canine diets.

## Data Availability Statement

The raw data supporting the conclusions of this article will be made available by the authors, without undue reservation.

## Ethics Statement

The animal study was reviewed and approved by University of Saskatchewan Animal Research Ethics Board.

## Author Contributions

CQ: formulated and created test diets, designed and performed experiments, and analyzed data and co-wrote the paper. YR: material preparation, starch analyses, data curation, validation, and review & editing. TM: performed experiments and review & editing. YA: Conceptualization, funding acquisition, investigation, project administration, resources, and review & editing. LW: Supervised animal research, experimental design, funding acquisition, investigation, resources, project administration, review & editing, and co-wrote the paper. All authors contributed to the article and approved the submitted version.

## Conflict of Interest

The authors declare that this study received funding or in-kind support from the Alberta Pulse Growers, Manitoba Pulse and Soybean Growers, Ontario Bean Growers, Saskatchewan Pulse Growers, Pulse Canada, Natural Sciences and Engineering Research Council (NSERC), Alliance Grain Traders (Saskatoon, SK Canada), Horizon Pet Foods (Rosthern, SK Canada) and Dr. Tom Warkentin (University of Saskatchewan, SK). The funders were not involved in the study design, collection, analysis, interpretation of data, the writing of this article or the decision to submit it for publication.
