# Supplementary material for: The Effects of 7 Days of Feeding Pulse-Based Diets on Digestibility, Glycemic Response and Taurine Levels in Domestic Dogs
Source: Front Vet Sci. 2021 May 5;8:654223. doi: 10.3389/fvets.2021.654223 (PMC8131660; doi:10.3389/fvets.2021.654223)
Supplement: Supplementary file 1 [file Data_Sheet_1.docx]

| Flour | Starch content (%, dry flour basis) *^c^* | Amylose content (%, dry flour basis) *^d^* | Amylose content (%, dry starch basis) *^e^* | Protein content (%, dry flour basis) *^f^* | Crude fiber content (%, dry flour basis) *^g^* | Fat content (%, dry flour basis) *^h^* | Ash content (%, dry flour basis) *^i^* | Dry matter (%) |
| --- | --- | --- | --- | --- | --- | --- | --- | --- |
| Rice | 86.5 ± 0.8 d | 21.9 ± 0.7 c | 25.3 ± 0.9 a | 7.3 ± 0.1 a | 0.02 ± 0.01 a | 0.74 ± 0.12 a | 0.48 ± 0.03 a | 88.1 ± 0.0 |
| Round pea (CDC Inca) | 48.0 ± 0.4 c | 19 ± 0.2 b | 39.5 ± 0.4 b | 23.9 ± 0.3 b | 5.64 ± 0.18 c | 1.23 ± 0.27 b | 2.35 ± 0.18 b | 92.6 ± 0.3 |
| Lentil (CDC Maxim) | 47.4 ± 0.6 c | 17.4 ± 0.7 ab | 36.8 ± 1.4 b | 27.6 ± 0.1 d | 3.55 ± 0.10 b | 0.39 ± 0.09 a | 2.41 ± 0.02 b | 93.6 ± 0.1 |
| Fava bean (CDC Snowdrop) | 43.1 ± 1.0 b | 16.4 ± 0.4 a | 38.0 ± 0.9 b | 30.3 ± 0.1 f | 7.98 ± 0.21 e | 0.69 ± 0.14 a | 3.02 ± 0.16 c | 93.2 ± 0.1 |
| Wrinkled pea (4140-4) | 34.1 ± 0.3 a | 24.1 ± 1 d | 70.6 ± 3 c | 28.7 ± 0.1 e | 7.79 ± 0.29 e | 1.82 ± 0.09 c | 3.11 ± 0.02 c | 92.7 ± 0.0 |
| Wrinkled pea (Amigold) | 34.4 ± 0.6 a | 27.1 ± 0.4 e | 78.8 ± 1.1 d | 26.4 ± 0.3 c | 7.00 ± 0.31 d | 1.90 ± 0.11 c | 3.40 ± 0.03 d | 92.8 ± 0.0 |

Table S1. Chemical compositions of flour samples *^a, b^*

*^a^* Values are presented as average ± standard deviation of triplicate measurements.

*^b^* Values followed by the same letter in the same column are not significantly different at *p* < 0.05.

*^c^* Determined using Megazyme Total Starch Assay Kit following AACC Method 76-13.01.

*^d^* Determined using an iodine colorimetric method of Chrastil (1987).

*^e^* Amylose content (%, dry starch basis) = [Amylose content (%), dry flour basis] / [Total starch content (%), dry flour basis] × 100.

*^f^* Determined using a Nitrogen/Protein Analyzer (CN628, LECO Corporation, St. Joseph, MI, USA), with a conversion factor of 6.25.

*^g^* Determined by Central Testing Laboratory Ltd. (Winnipeg, Manitoba, Canada), following Crude Fiber Method by Ankom Technology (2017).

*^h^* Determined by Central Testing Laboratory Ltd. (Winnipeg, Manitoba, Canada), following AOCS Method Am 5-04.

*^i^* Determined by Central Testing Laboratory Ltd. (Winnipeg, Manitoba, Canada), following AOAC Method 942.05.

| Parameters | Condition |
| --- | --- |
| Feed Moisture (%) | 30 |
| Solid Feed Rate (kg/h) | 3.2 |
| Screw Speed (rpm) | 200 |
| Temperature (Section 1, ˚C) | 40 |
| Temperature (Section 2, ˚C) | 60 |
| Temperature (Section 3, ˚C) | 80 |
| Temperature (Section 4, ˚C) | 100 |
| Temperature (Section 5, ˚C) | 120 |
| Die Diameter (mm) | 5.5 |

Table S2: Extruder parameters used to create test diets
